# Activated carbons modified by magnesium oxide as highly efficient sorbents for acetone†

**DOI:** 10.1039/c7ra11740j

**Published:** 2018-01-15

**Authors:** Ke Zhou, Liqing Li, Xiancheng Ma, Yamian Mo, Ruofei Chen, Hailong Li, Haoyang Li

**Affiliations:** School of Energy Science and Engineering, Central South University Changsha 410083 Hunan China liqingli@hotmail.com +86 13807483619; School of Materials Science and Engineering, Central South University Changsha 410083 Hunan China

## Abstract

Porous activated carbon modified with MgO was synthesized by an evaporation-induced self-assembly (EISA) method for its application to acetone capture. The textural and chemical characteristics of five modified activated carbon composites (AC–MgO) were characterized using X-ray diffraction, scanning electron microscopy, transmission electron microscopy and nitrogen adsorption isotherm measurements. The adsorption behaviors of samples for acetone were investigated and correlated to their physical and chemical properties. Density functional theory was also employed to calculate the charge transfer, the equilibrium distance, and the adsorption energy of acetone adsorbed on a carbon surface functionalized with crystalline MgO. An AC–MgO-10% sample with balanced surface area, microporosity and MgO content exhibited the highest acetone adsorption capacity (432.7 mg g^−1^). The results indicate that an appropriate MgO content on AC can effectively improve the adsorption capacity of acetone ascribed to strong chemisorption between MgO nanoparticles and acetone molecules.

## Abstract 2

Porous activated carbon modified with MgO was synthesized by an evaporation-induced self-assembly (EISA) method for its application to acetone capture.

## Graphical abstract

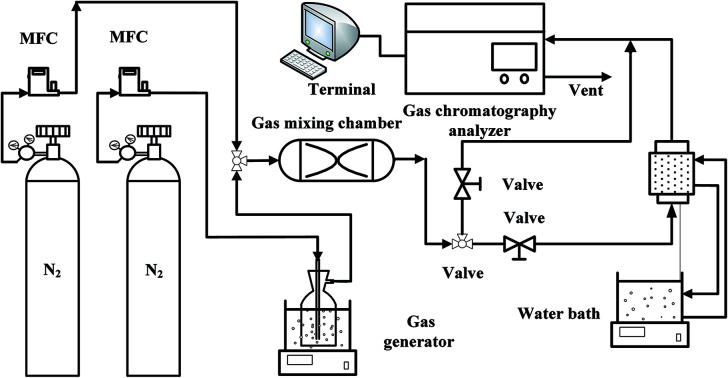

## 1. Introduction

Volatile organic compounds (VOCs), the principle causes of severe air pollution, are often malodorous, toxic and carcinogenic even at a very low concentration.^1–3^ Therefore, the problem of how to remove and dispose of VOCs has attracted extensive attention from society. At present, several methods including condensation, distillation, biological treatment, catalytic oxidation and adsorption have been used for VOC abatement.^4–8^ Among them, adsorption is considered as the most economic and effective technique for control over a variety of VOCs, while an appropriate adsorbent is the key to adsorption techniques.^9,10^ Up to now, various adsorbents derived from silica, zeolites, metal organic frameworks, metal oxides and activated carbon have been employed to capture VOCs,^11–16^ and activated carbon is regarded as one of the most promising adsorbents for commercial use owing to its great surface area, excellent thermal/chemical stability, low cost and outstanding cyclic utilization.

On the other hand, carbon-based porous materials are endowed with favorable surface hydrophobicity due to the high density of carbon atoms in graphite-like sheets, which is greatly beneficial to VOCs absorption.^17^ In general, activated carbon with slit-shaped micropores is associated with a high physical adsorption capacity for multifarious VOCs in a highly dispersive way.^18^ However, the widespread use of activated carbon has also deprived it of the ability to trap a specific gas for instance acetone, one of oxygenated volatile organic compounds (OVOCs). Meanwhile, chemical adsorption is an essential factor to be considered apart from physical adsorption. Researchers have demonstrated that metal oxide materials have an excellent adsorption ability for OVOCs.^19^ MgO, an alkali earth metal oxide, is an outstanding adsorbent for OVOCs adsorption under normal temperature and pressure.^20,21^ Nonetheless, MgO prepared by thermal decomposition of magnesium acetate has low surface area, which is a big challenge for its applications.^22^ Thus, improvements in active surface area, accessibility to adsorption sites and synergistic porous support are of great significance to the practical applications of MgO.

Recently, porous carbon and metal oxide composites have been developed with metal oxide nanoparticles doped into the matrix of nanoporous carbon materials,^21,23–25^ which remarkably improves the adsorption capacity towards VOCs compared to the single-phase activated carbon materials and thus attracts extensive attention. The above-mentioned porous carbon/metal oxide composites are endowed with excellent physical adsorption attributed to the large surface area of activated carbon. Furthermore, they also show outstanding chemical adsorption ability due to a large number of active adsorption sites in the metal oxide. To the best of our knowledge, there is rare literature concerning the adsorption capacity of activated carbon and MgO composites materials for acetone.

In this study, MgO-doped porous carbon materials were successfully prepared through a facile evaporation doping method, with magnesium acetate being used as the Mg resource while commercial activated carbon (AC) as the porous carbon frameworks for the special support for MgO. The resulting AC–MgO samples were subsequently characterized *via* BET surface area, X-ray diffraction (XRD) and scanning electron microscope (SEM) analyses, and their adsorption performances towards acetone were also examined through dynamic adsorption tests. More importantly, the synergistic effect of nanosized MgO-doped porous carbon on its acetone adsorption performance was investigated using quantum chemical molecular modeling study.

## 2. Experimental

### 2.1 Materials

All chemicals were obtained from commercial sources and used without further purification. Magnesium acetate tetrahydrate (C_4_H_6_O_4_Mg·4H_2_O, 98%), acetone (CH_3_COCH_3_, 99.5%), ammonia solution (NH_4_OH, 33%) and ethanol (C_2_H_5_OH, 99.7%) were purchased from Xilong Chemicals Co., Ltd., China. The commercial activated carbon was provided by Changge Henan Limin Activated Carbon Co., Ltd., China. Nitrogen (N_2_) gas of ultrahigh purity (99.999%) was purchased from High-tech Gas Co., Ltd., China.

### 2.2 Preparation of samples

The activated carbons were modified by deposition of MgO nanoparticles *via* a combination of evaporation induced self-assembly process with subsequent pyrolysis treatment. In a typical synthesis, 2.35 g of magnesium acetate was first dissolved in 100 mL of distilled water, and then 4.00 g of activated carbon was dropped slowly into this solution with magnetic stirring at room temperature overnight. The mixture was evaporated at 373 K and then dried at 393 K to prepare the impregnated sample. Subsequently, the impregnated sample was heated to 823 K (5 K min^−1^) under N_2_ flow (100 mL min^−1^) for 4 h in a tubular furnace. Thus AC–MgO composites were obtained, which are denoted as AC–MgO-10% as the MgO content is 10%. Other MgO–AC-*n*% samples were prepared using the same method but with different MgO content. As a result, four AC–MgO composite samples were obtained and denoted as AC–MgO-5%, AC–MgO-10%, AC–MgO-20%, AC–MgO-30% respectively. For comparison, the pure MgO was synthesized by thermal decomposition of magnesium acetate at 823 K under N_2_ flow for 4 h.

### 2.3 Characterization

The samples were determined by XRD (X′PertPro MPD, PANalytical B.V., NED) with a PANalytical powder diffractometer operated at 40 mA and 40 kV using Cu/Kα as the radiation source. The scanning range was from 5° to 80° (2*θ*) with a scanning step of 0.02° per second. The sizes and morphologies of AC–MgO samples were studies through SEM (Helios NanoLab 600i, FEI Co., USA), where the measurements were operated under 10–20 kV acceleration voltage. Transmission electron microscopy (TEM) images were performed using a Tecnai G2 20S-Twin electron microscope equipped with a cold field emission gun under an acceleration voltage of 200 kV. The microporosity characterization of AC–MgO composites were determined by N_2_ adsorption at 77 K with a specific surface area analyzer (JWGB Sci. &Tech. Co., Ltd., China). The samples were ground to powders and degassed at 373 K for 4 h in a vacuum before the test. The Brunauer–Emmett–Teller surface area (*S*_BET_), micropore volume (*V*_m_) and pore size distribution (PSD) of the as-synthesized samples were calculated from the N_2_ isotherm by the Brunauer–Emmett–Teller (BET) equation,^26^*t*-plot method,^27^ Horvath–Kawazoe (HK) equation,^28^ and Barrett–Joyner–Halenda (BJH) method^29^ respectively. The total pore volume (*V*) was estimated from the volume of N_2_ (as liquid) held at a relative pressure of 0.98.

### 2.4 Acetone adsorption experiments

**Fig. 1:**
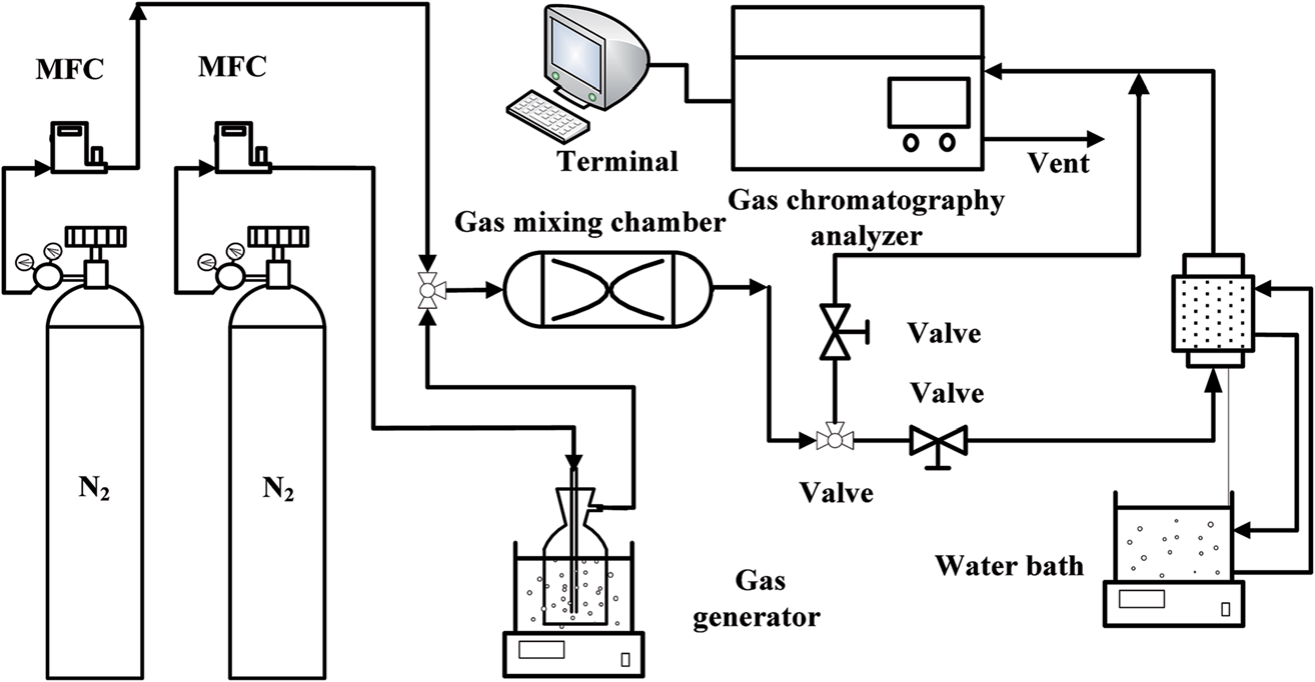
Isothermal adsorption device for acetone adsorption.

The experimental system of acetone adsorption is shown in Fig. 1. Acetone breakthrough experiments were carried out in a glass tubular column equipped with a water jacket to maintain a constant temperature at 298 K. The nitrogen gas system consists of two parts and controlled by mass flow controller (D08-3F, Sevenstar Co., Ltd., China), one branch was passed through acetone gas generator to obtain high concentration vapor at 273 K. At the same time, in order to regulate the concentration of acetone vapor, nitrogen gas got to the gas mixing chamber by the other branch to dilute the high concentration acetone vapor. Subsequently, the gaseous mixture was directed onto the fixed-bed of a sorbent material at constant temperature 298 K and atmospheric pressure. The output concentration of the fixed-bed was monitored online by a gas chromatograph (GC) (SP-6890, Ruihong Chemical, Co., Ltd., China), connected directly to the adsorption column outlet.

### 2.5 Computational details

All the first principle calculations were performed using density functional theory (DFT) method in DMol^3^ software of Materials Studio package.^30^ The generalized gradient approximation (GGA) with the Perdew–Burke–Ernzerhof (PBE) exchange–correlation functional was used to model the electron exchange and correlation interaction.^31^ Core treatment of the metal atoms were described by the density functional semicore pseudopotentials (DSPP),^32^ which was developed specifically for DMol^3^ calculations. All electron double numerical atomic orbitals augmented by p- and d-polarization functions (DNP) basis set was used to describe the atomic orbital.^33^ The thermal smearing level was set at 0.005 to improve the convergence, and a real-space orbital global cutoff of 3.7 Å was applied, and the convergence threshold parameters for the optimization were 10^−5^ (energy), 2 × 10^−3^ (gradient), and 5 × 10^−3^ (displacement), respectively. This basis sets are similar to 6-31G (d, p) in size, while they tend to be more accurate and have little basis set superposition error (BSSE).^33^ BSSE was not taken into account for calculating the energies, as the numerical basis sets implemented in DMol^3^ minimized or even eliminated basis set superposition error.^34^ Calculations were carried out in a model system containing an acetone molecule adsorbed on the (100) face of MgO represented by a cluster of 32 O atoms and 32 MgO atoms, (*i.e.* with the (MgO)_32_ formula).^35^ Because of the complexity and uncertainty of the activated carbon structure, it was modeled as monolayer graphene slabs in 7 × 7 carbon ring unit cells (98 atoms for pure graphene).^36,37^ The adsorption behavior of acetone onto the surface of respective substrate was simulated, and the acetone adsorption energy was calculated in the following manner:

1 *E*_ad_ = *E*_surface+acetone_ − (*E*_surface_ + *E*_acetone_)

where *E*_ad_, *E*_surface+acetone_, *E*_surface_ and *E*_acetone_ are adsorption energy, total energy of adsorbate–surface complex, surface of substrate and isolated acetone, respectively. The negative value of *E*_ad_ indicates that the adsorption is an exothermic reaction, and a higher negative value of *E*_ad_ corresponds to a more stable structure and stronger interaction.

## 3. Results and discussion

### 3.1 XRD pattern of samples

**Fig. 2:**
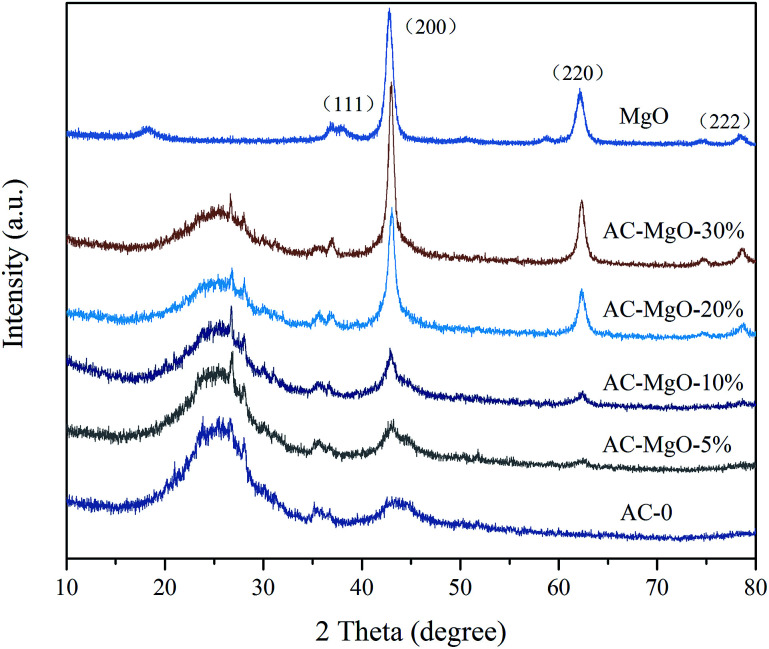
XRD patterns of pure MgO and AC–MgO composites with different MgO content.

XRD pattern of the original activated carbons (AC-0), AC–MgO composites and pure MgO is shown in Fig. 2. From the diagram, all the samples except pure MgO display two broad peaks at around 25° and 44°, which were assigned to the characteristic carbon (100) and (101) diffractions respectively.^38^ A large intensity increase in the low-angle scatter shows the presence of a high density of nanopores. Compared to the original activated carbon (AC-0), the crystalline cubic structure of MgO appeared on the patterns of the AC–MgO composites and pure MgO, with the 2 theta values of 36.8°, 42.9°, 62.3° and 78.5° corresponding to the (111), (200), (220) and (222) planes, respectively.^39^ It is worth noting that these four peaks became obviously stronger; meanwhile, their full width at half maximum (FWHM) was marginally reduced as the MgO content in the AC–MgO composites increased. Additionally, no diffraction peaks of impurities could be observed for all the samples, which further confirm the magnesium acetate tetrahydrate was fully decomposed at 823 K and completely converted to MgO. Meanwhile, the average crystallite size of MgO nanocrystal was calculated using the Scherrer equation,^40,41^ and the Scherrer equation is expressed as follows:2
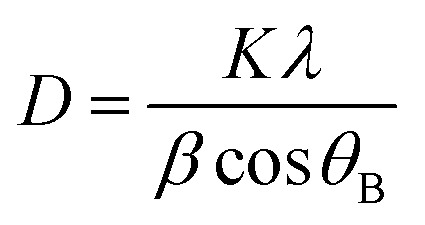
where *D* is the average size of the ordered (crystalline) domains, *K* is the shape factor with a typical value of about 0.9, *λ* is the X-ray wavelength, *β* is the full width at half maximum of the reflection peak, and *θ*_B_ is the Bragg angle. The average MgO crystallite of AC–MgO composite is 25.6 nm.

### 3.2 Morphology analysis

**Fig. 3:**
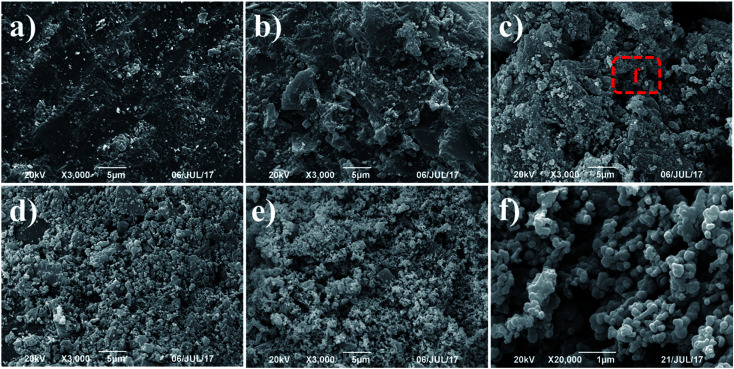
SEM images of AC–MgO composites with different MgO content: (a) AC-0, (b) AC–MgO-5%, (c) AC–MgO-10%, (d) AC–MgO-20%, (e) AC–MgO-30% and (f) the amplified SEM image of AC–MgO-10%.

SEM technique was used to observe the surface physical morphology of the as-obtained AC–MgO composites with different MgO content. The micrographs are given in Fig. 3. In the case of AC (see Fig. 3a), the skeleton of the carbon monolith did not have defect or crack on the surface of unmodified AC samples. However, it was observed that the synthesized AC–MgO (see Fig. 3b–e) collapses into small pieces and form a large number of through holes as a result of the high temperature activation process. Furthermore, with the increase of the impregnation concentration, more and more magnesia particles were supported on the surface of activated carbon. It can be clearly seen that the surface of the AC becomes coarse as the amount of MgO increases. Referring to the amplified SEM image in Fig. 3f, the MgO particles of composites surface presented a disordered small spherical structure and overlap with each other to generate a “grape” morphology. This fact implies that with the increase of the calcination temperature, the particle size grew up due to the particle aggregation at high calcination temperature.

**Fig. 4:**
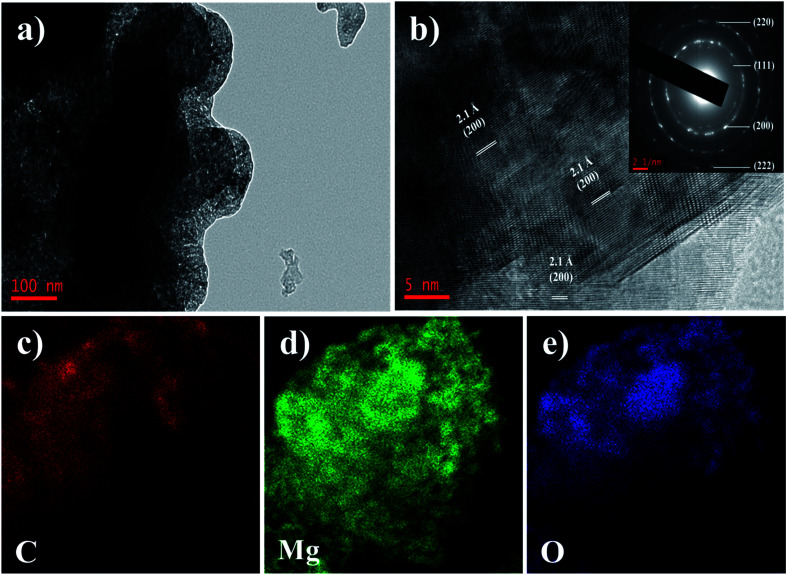
(a) TEM and (b) HR-TEM images of the AC–MgO-10% sample, and the inset of (b) is the corresponding SAED patterns. (c–e) EDS mapping elements of carbon, magnesium and oxygen of the AC–MgO-10% sample.

To further reveal the microstructure of the AC–MgO composites, TEM, selected area electron diffraction (SAED) and energy dispersive spectroscopy (EDS) measurements were performed. Since all samples have similar morphology, the AC–MgO-10% sample was chosen as a typical example. The TEM image (see Fig. 4a) displays that the sample consisted of many small MgO nanocrystals and carbon matrix. Fig. 4b shows the HR-TEM image of AC–MgO-10%. MgO crystal lattice fringes with an interlayer spacing of 2.1 Å were attributed to the (200) lattice planes of cubic MgO. The SAED patterns of the corresponding samples (inset of Fig. 4b) shows bright circled rings, indicating the polycrystalline nature of MgO in the selected area. The diffracting concentric rings assigned to (111), (200), (220) and (222) reflections of MgO are evident, and the result is consistent with the MgO planes obtained from XRD data. As shown in Fig. 4c–e, EDS mapping results demonstrate the Mg and O elements was distributed on the activated carbon surface uniformly rather than apparent aggregation.

### 3.3 Nitrogen adsorption isotherms

**Fig. 5:**
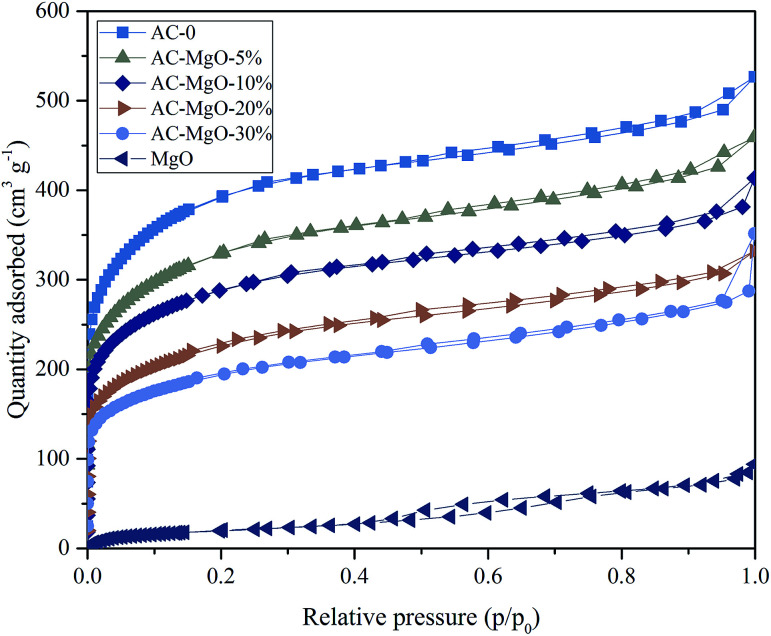
N_2_ sorption isotherms of pure MgO and AC–MgO composites with different MgO content.

To investigate the porous structure of the AC–MgO composite materials, the surface area and pore size analysis were carried out. Fig. 5 shows the nitrogen adsorption–desorption isotherms of the samples. All the composite and carbon materials exhibit representative type-IV isotherm profiles with unusual small H4 hysteresis loops in the relative pressure range of 0.4–1.0 according to the International Union of Pure and Applied Chemistry (IUPAC) classification. The type H4 hysteresis loop was observed, with the characteristic step down at medium relative pressures (*P*/*P*_0_ ≈ 0.4). This kind of hysteresis loop appeared as the complex materials contain both micropores and mesopores, and the phenomenon of capillary condensation occurred in mesopores.^42^ Actually they presented a narrow pore-size distribution centered at 0.5–2.5 nm. It is worth noting that the nitrogen uptake of all samples increased sharply at low relative pressure (*P*/*P*_0_ ≈ 0.01), indicating the presence of abundant micropores in these porous composite materials.^43,44^ However, it can be seen from Fig. 5 that pure MgO samples showed an isotherm of type-II with a H4 hysteresis loops in the relative pressure range of 0.4–0.8, which implied the existence of mesoporous structure in MgO.

**Fig. 6:**
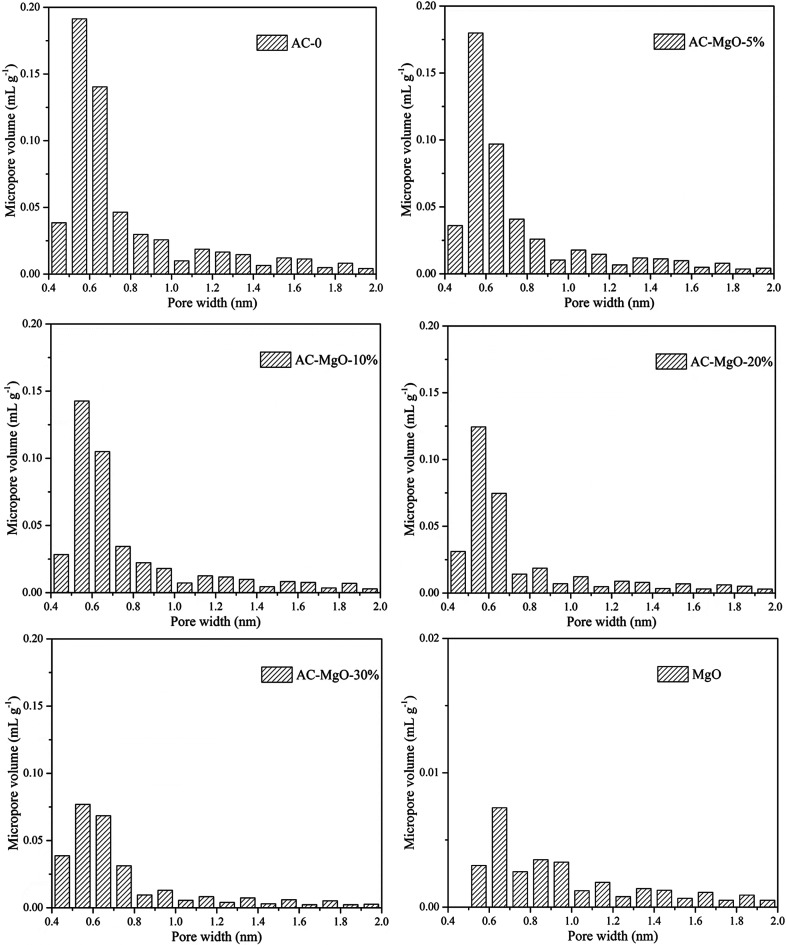
Pore size distribution of pure MgO and AC–MgO composites with different MgO content.

In addition, the pore size distributions of all samples are shown in Fig. 6. The activated carbon modified by MgO did not have significant differences in the pore size distributions at different MgO content. From Fig. 6, a range of 0.4–1.0 nm was observed as the concentration area of micropore distribution, which accounts for more than 81% of the total, with an obvious bulge in the micropore volume at 0.5–0.7 nm. This also confirms the previous argument that these porous materials are mainly composed of microporous structure. However, with increasing MgO content, the micropore volume decreased slightly. The decrease in microporosity can be explained as a result of blockage from MgO nanoparticles deposition on pore walls. The micropore size distribution remained about the same at 0.5–0.7 nm after modification, indicating that the deposition of metal oxides and heat treatment only affects the size of the microporous structure without generating a new porous structure. It can be seen from Fig. 6 that the pure MgO samples have almost no micropores.

The detailed information of surface area and pore size of all the synthesized samples is summarized in Table 1. A similar trend of the surface area and the total pore volume could be observed, which decreased with rising MgO content (0–30%). Clearly, the original AC shows the maximum surface area of 1464 m^2^ g^−1^ and pore volume of 0.81 mL g^−1^. In contrast, the synthesized AC–MgO-*n*% samples have surface area of 713–1213 m^2^ g^−1^ and pore volume of 0.54–0.71 mL g^−1^, which are much lower than the unmodified AC samples. It is obvious that with the enrichment of MgO on AC, more and more MgO nanoparticles blocked the micropores of activated carbon. However, the volume of mesopores and macropores did not change much with the MgO content. Consequently, a large quantity of MgO species particle aggregate at micropores rather than mesopores and macropores.

**Table 1:** Porous structure parameters of the pure MgO and AC–MgO composites

Samples	*S*_BET_ m^2^ g^−1^	*S*_mic_ m^2^ g^−1^	*S*_mes_ + *S*_mar_ m^2^ g^−1^	*V*_total_ mL g^−1^	*V*_mic_ mL g^−1^	*V*_mes_ + *V*_mar_ mL g^−1^	MgO wt%
AC	1464	1076	388	0.81	0.58	0.23	0%
AC–MgO-5%	1213	837	376	0.71	0.48	0.23	5%
AC–MgO-10%	1067	758	309	0.64	0.43	0.21	10%
AC–MgO-20%	823	532	291	0.61	0.33	0.28	20%
AC–MgO-30%	713	463	250	0.54	0.28	0.26	30%
MgO	120	25	95	0.15	0.03	0.012	100%

### 3.4 Acetone adsorption study

**Fig. 7:**
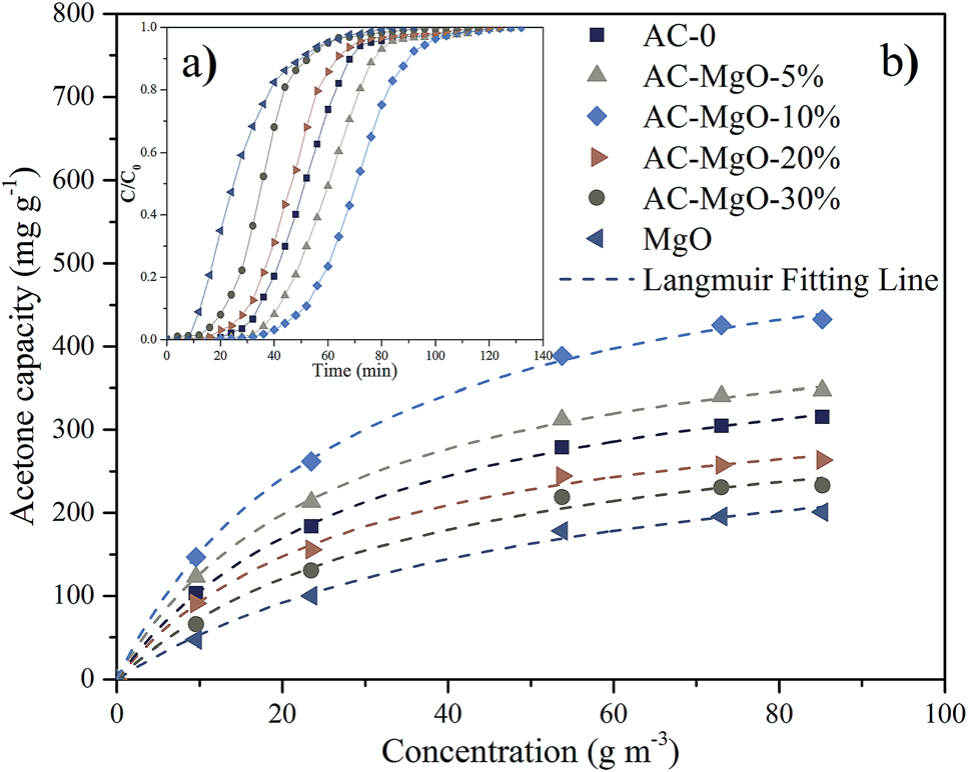
(a) Breakthrough curves and (b) adsorption isotherms of acetone on pure MgO and AC–MgO composites with different MgO content.

The typical results of acetone adsorption were shown in Fig. 7a as breakthrough curves. In consideration of the diversity in absolute concentrations, the concentrations presented were then normalized, ranging from 0 to 1. The adsorption capacity was assessed by numerical integration of the region calculated from the acetone breakthrough curve. Notably, the outlet acetone concentration was zero in all the adsorption experiments, indicating a thorough acetone removal before the breakthrough. The amount of sample used in each experiment was 0.2 g.

As shown in Fig. 7a, the development trend of the six penetration curves was consistent at a certain temperature (298 K) and concentration (85.21 m^3^ g^−1^). Specifically, the curve started very gently, while the concentration increased dramatically as soon as the breakthrough occurred. With the passage of time, a gradual stabilization in concentration was observed. The adsorption capacity of AC–MgO-10% ranks the first among all the samples. Breakthrough time is defined as time when outlet concentration was 10% of inlet concentration.^45^ The breakthrough time was also different for each sample, and the breakthrough time of acetone in AC–MgO-10% is the longest, suggesting its superior acetone capture capacity.

Here, the Langmuir equation, which considers monolayer adsorption on a homogeneous surface with negligible intermolecular force,^46^ was used to fit the adsorption isotherms of acetone on the samples, and the Langmuir equation is expressed as follows:3
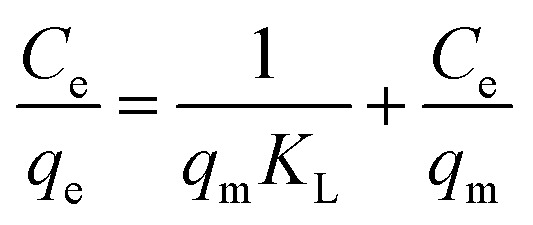
where *C*_e_ (g m^−3^) refers to the equilibrium concentration, *q*_e_ (mg g^−1^) is the amount of adsorbate on per unit weight of adsorbent, *q*_m_ (mg g^−1^) is the monolayer adsorption capacity, and *K*_L_ (m^3^ g^−1^) is a constant related to adsorption rate. The fitting results are listed in Table 2.

**Table 2:** Langmuir equation fitting of acetone adsorption

Sample	*q*_m_ (mg g^−1^)	*K*_L_ (m^3^ g^−1^)	*R* ^2^
AC-0	433.86	0.0322	0.9996
AC–MgO-5%	460.75	0.0377	0.9995
AC–MgO-10%	586.22	0.0351	0.9994
AC–MgO-20%	359.88	0.0342	0.9971
AC–MgO-30%	349.09	0.0265	0.9936
MgO	336.87	0.0187	0.9948

As shown, the experimental data were consistent with Langmuir equation. By varying the acetone concentration (9.56–85.21 g m^−3^) at a fixed temperature (298 K), a set of adsorption isotherms were obtained for all samples (shown in Fig. 7b). According to the linear regression method, the Langmuir equations are suitable for the adsorption of acetone (*R*^2^ > 0.993). Apparently, type-I profiles were observed in all adsorption isotherms of acetone in accordance with IUPAC classification, in agreement with the microporous structure of porous carbons. The adsorbed amount of acetone on samples increased rapidly at the initial stage, and then kept a temperate rise with increasing adsorption concentration. By comparison, the parameter *q*_m_ of the Langmuir model fitting was larger than adsorption capacity of the experiment, and the numerical order of *q*_m_ was consistent with the order of the experiment. The possible explanation for this difference is that the *q*_m_ refers to the maximal amount of adsorption corresponding to a complete monolayer coverage on the surface, while the experiment does not necessarily reach this value.

To further evaluate the adsorption performance, we compared the acetone uptake values between the samples synthesized in this work and the previously reported adsorbents, and the detailed experimental data are listed in Table 3. It can be seen that the acetone adsorption capacity of AC–MgO-10% was much higher than those of conventional adsorbents, indicating that the AC–MgO-10% is a promising adsorbent used for acetone removal.

**Table 3:** Equilibrium amount of acetone adsorbed on various adsorbents

Adsorbent	*Q*_e_ (mg g^−1^)	Temperature (K)	Inlet concentration (g m^−3^)	Reference
BASF AC	360.1	298	468.85	47
MSPs	141.1	318	22.53	48
Si-MSM-41	157.6	318	22.53	48
DAY-zeolite	136.8	293	548.03	49
Commercial AC	293.4	293	50.00	50
AC–FA	327.1	283	83.91	51
AC–OA	310.2	283	83.91	51
AC–SA	298.1	283	83.91	51
AC–MgO-5%	347.0	298	85.21	This work
AC–MgO-10%	433.0	298	85.21	This work

**Fig. 8:**
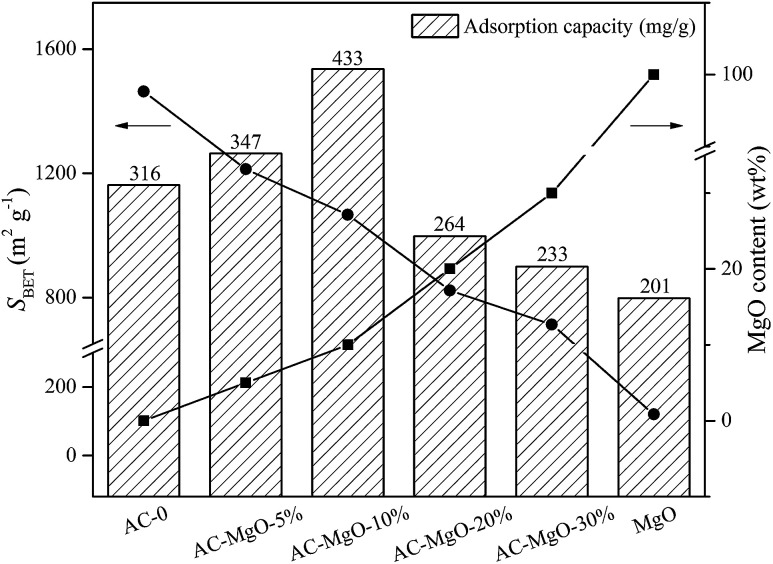
The adsorption capacity of samples and its relationship with the surface area and MgO content.

In addition, we compared the abilities of pure MgO and AC–MgO composite samples in acetone capture, which presented with diverse surface area and MgO content (at 298 K, 85.21 m^3^ g^−1^ acetone) (see Fig. 8). Consequently, it varied significantly in view of the capacities of acetone adsorption from six samples under certain situations.

There is no doubt that the presence of MgO species as well as the surface area is actively involved in the determination of capacity of acetone adsorption. Of note, there is a decline in surface area along with an elevation in MgO content. This is due to the gradual block of the pore in the activated carbon attributed from the increase in the number of MgO nanoparticles. As shown in Fig. 8, we compared the adsorption capacity of AC–MgO composites and unmodified activated carbon. As a result, the AC-0 sample presented with a capacity of acetone adsorption at 315 mg g^−1^, and the AC–MgO-10% sample ranked first in capacity of acetone uptake at 433 mg g^−1^, which harbored intermediate surface area (1067 m^2^ g^−1^) as well as MgO content (10 wt%). In consideration of the Lewis-base active sites provided by MgO nanoparticles,^52,53^ which play a vital role in binding the acidic acetone, the advantage of higher MgO content is more obvious even if the specific surface area of AC–MgO-10% is smaller than that of AC-0. However, the pure MgO sample, with the highest MgO content (100 wt%) but the smallest surface area (120 m^3^ g^−1^), displayed the lowest acetone uptake capacity at 201 mg g^−1^. The reason is that the surface area of pure MgO is too small to expose enough active sites of MgO surface. The above finding indicates that the surface area is also involved in acetone adsorption. Collectively, the acetone adsorption capacity of AC modified by MgO depends on the MgO content as well as the surface area.

### 3.5 Molecular modeling study

**Fig. 9:**
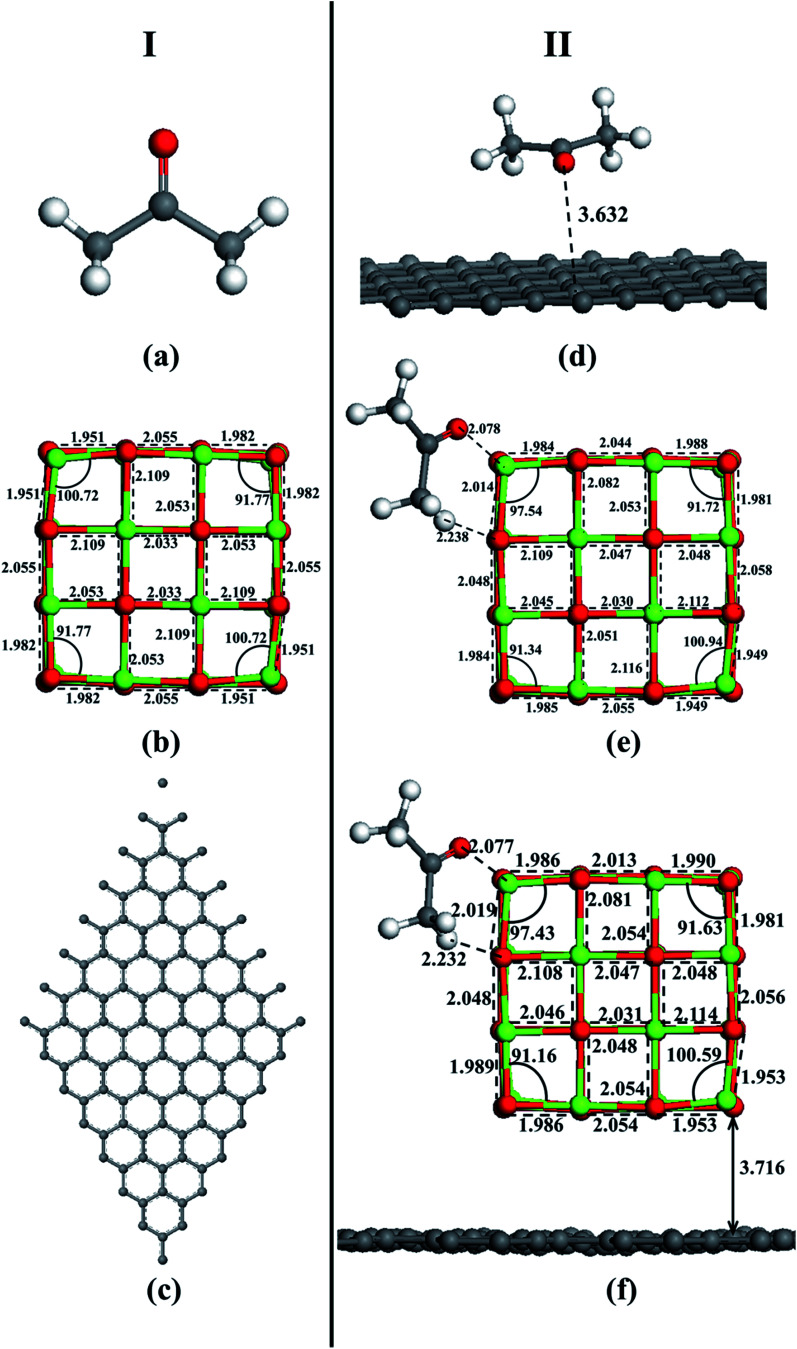
Optimized structures of (I): (a) acetone, (b) MgO and (c) graphene, and (II): (d) acetone–graphene, (e) acetone–MgO, (f) acetone–MgO/graphene.

In order to prove that the acetone adsorption on MgO-doped activated carbon samples is not only the physical adsorption but also chemical adsorption tendency, the adsorption of acetone molecules on the surface of activated carbon, MgO and MgO–graphene composites was simulated using DFT method. After fully optimization by the GGA-PBE method and considering all the spatial positional relationships between adsorbate and adsorbent, optimized structures were obtained (see Fig. 9). The result of molecular interaction of acetone with graphene surface is shown in Fig. S1.† Obviously, interaction between acetone and graphene is very weak even in different adsorption modes. However, from Fig. S2,† molecular interaction of acetone with MgO surface, we can see that O and H atoms in acetone interact more easily with Mg and O atoms on the edge sites at MgO surface, respectively, which is the best adsorption mode. The surface reactivity of the MgO strongly depends to the site selected for the molecule adsorption. The MgO face without defects has a low reactivity producing a weak adsorption but the adsorption on steps, edges and corners is stronger.^54^ The same applies to acetone–MgO/graphene system (see Fig. S3†).

After geometry optimization of the functionalized carbon surface, acetone adsorption energy on the surface was calculated. The values of adsorption energy for acetone complexes with surface models are given in Table 4. The greater the negative value of adsorption energy, the stronger the adsorption capacity is.^55^ As shown, acetone molecule exhibits the stronger interaction with pure MgO with the energy value of −107.89 kJ mol^−1^, which is categorized in the region of chemisorption, while the values of adsorption is given −4.20 kJ mol^−1^ for pristine graphene, which is categorized at physisorption region.^56^ This can be attributed to the pure MgO nanoparticles providing the active adsorption sites of Lewis base for acidic gas acetone.^57^ Notably, the highest binding energy occurs in the acetone–MgO/graphene system, −118.13 kJ mol^−1^, and the energy value is larger than the sum of the other two systems. As suggested by the results, the interaction between MgO and graphene can promote the adsorption of acetone.

**Table 4:** The charge transfer (*Q*), the equilibrium distance (*d*_e_), and adsorption energy (*E*_ad_) of all systems

System	*Q* (e)	*d*_e_ (Å)	*E*_ad_ (kJ mol^−1^)
Acetone–MgO	−0.201	2.078	−107.89
Acetone–graphene	−0.005	3.632	−11.55
Acetone–MgO/graphene	−0.207	2.077	−118.13

**Fig. 10:**
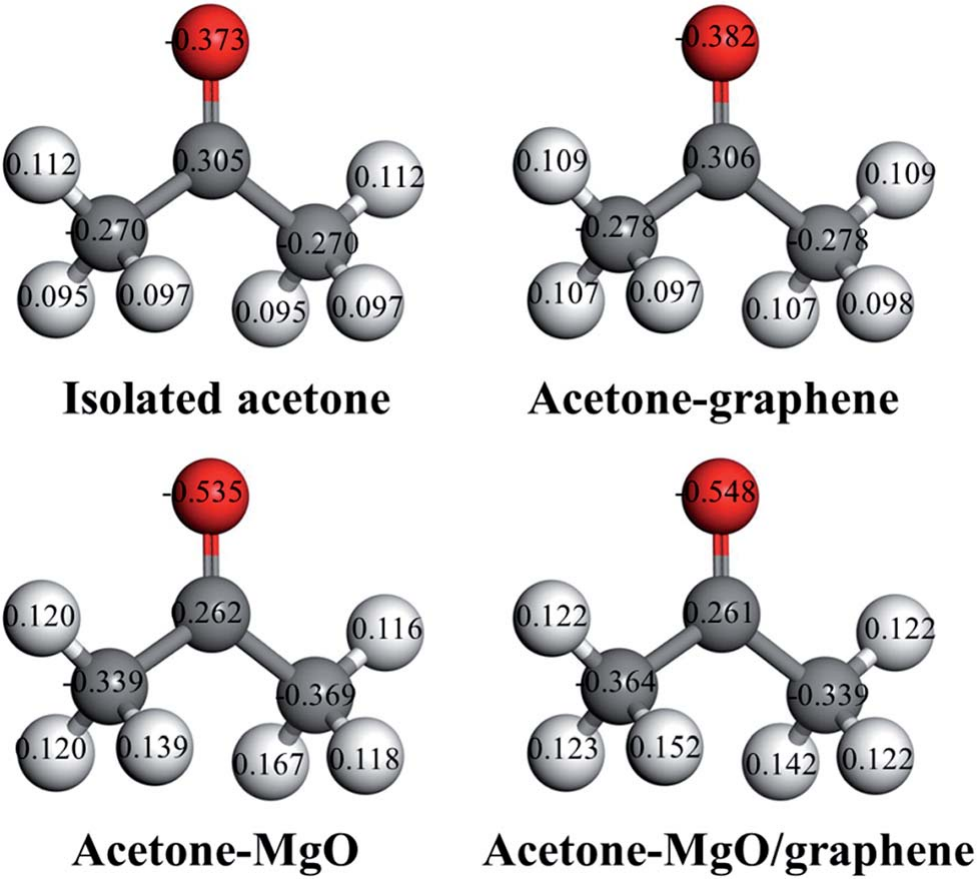
Local charge distribution of acetones molecule before and after adsorption interaction with MgO, graphene and MgO/graphene.

The local charge distribution of acetone molecule before and after adsorption has been calculated with the GGA-PBE method so as to confirm the Lewis acid–base interaction between acetone and MgO nanoparticles (Fig. 10). Through comparing the charge distribution of separate acetone molecule with its complexes, it can be found that the electronic structure of acetone molecule is subjected to charges upon adsorption. As is shown in Fig. 10, acetone molecule carries the highest negative charges in the electronic structure after interacting with MgO–graphene model. The net charges are transferred from the surface of MgO–graphene (the electron pair donor) to acetone molecule (the electron pair acceptor), which results in a sharp increase in the charges of acetone molecule, as can be observed from net charge transfer data in Table 4.

Another important index to measure the strength of adsorption is the adsorbate–adsorbent equilibrium distance. Generally speaking, the shorter distance is linked with stronger interaction.^58^ It can be seen in Fig. 9 that, acetone–MgO/graphene (2.077 Å) achieves the shortest equilibrium distance, which is much longer in acetone–graphene (3.632 Å). These simulation results are totally in agreement with experiment results. From the results of experiment and simulation, it can be concluded that MgO modified activated carbon is an effective method for increasing the adsorption of acetone.

## 4. Conclusions

In summary, a facile and controllable method has been developed to prepare porous activated carbons modified by MgO nanocomposites using magnesium acetate as magnesium source *via* a combination of evaporation induced self-assembly process with subsequent pyrolysis treatment. The optimized AC–MgO-10% composites exhibits remarkable acetone captures capacity up to 433 mg g^−1^ (at 298 K, 85.21 m^3^ g^−1^ acetone). Notably, increasing MgO content will greatly enhance the chemical adsorption of acetone on the composite but in the meantime decrease the physical adsorption. Quantum chemical calculation reveals that the electronic properties of MgO/graphene composites are responsible for its high adsorption capacity towards acetone and the interaction belongs to chemisorption. This simple synthesis strategy can be extended to other metal oxide/carbon composites that hold great promise for OVOC adsorption.

## Conflicts of interest

There are no conflicts to declare.

## Supplementary Material

RA-008-C7RA11740J-s001
