# Supplementary material for: Activated carbons modified by magnesium oxide as highly efficient sorbents for acetone
Source: RSC Adv. 2018 Jan 15;8(6):2922–32. doi: 10.1039/c7ra11740j (PMC9077576; doi:10.1039/c7ra11740j)
Supplement: RA-008-C7RA11740J-s001 [file RA-008-C7RA11740J-s001.pdf]

Electronic Supplementary Material (ESI) for RSC Advances.
This journal is © The Royal Society of Chemistry 2018

Supporting information

Activated carbons modified by magnesium oxide as highly efficient sorbents for acetone

Ke Zhou [a], Liqing Li [a, *], Xianchen Ma [a], Yamian Mo [a], Ruofei Chen [a], Hailong Li [a], Haoyang Li [b]

[a] School of Energy Science and Engineering, Central South University, Changsha 410083, Hunan, China
[b] School of Materials Science and Engineering, Central South University, Changsha 410083, Hunan, China
* Corresponding author. Tel: +86 13807483619, E-mail address: liqingli@hotmail.com

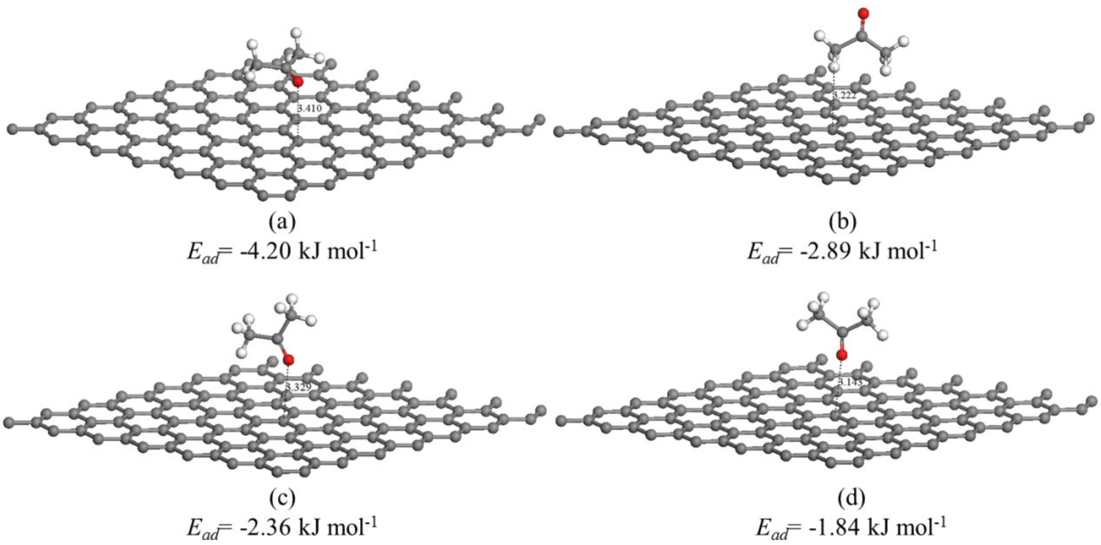

Figure S1. Molecular interaction of acetone with graphene surface.

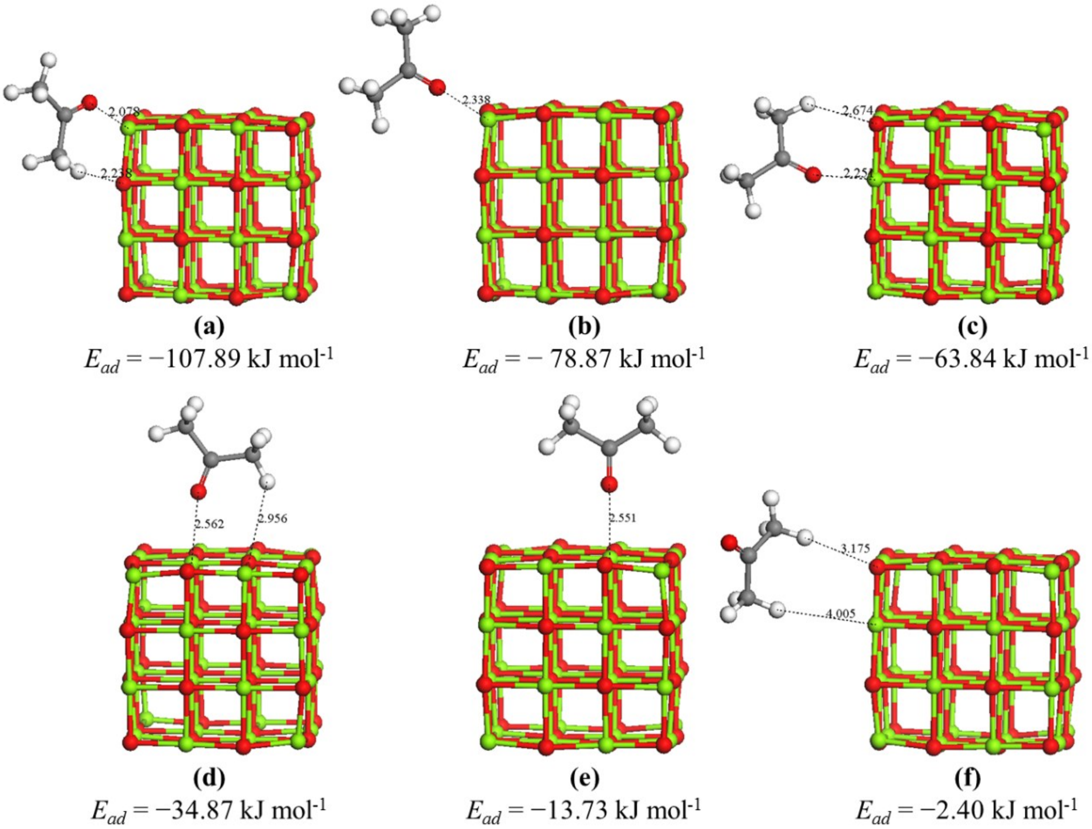

Figure S2. Molecular interaction of acetone with MgO surface.

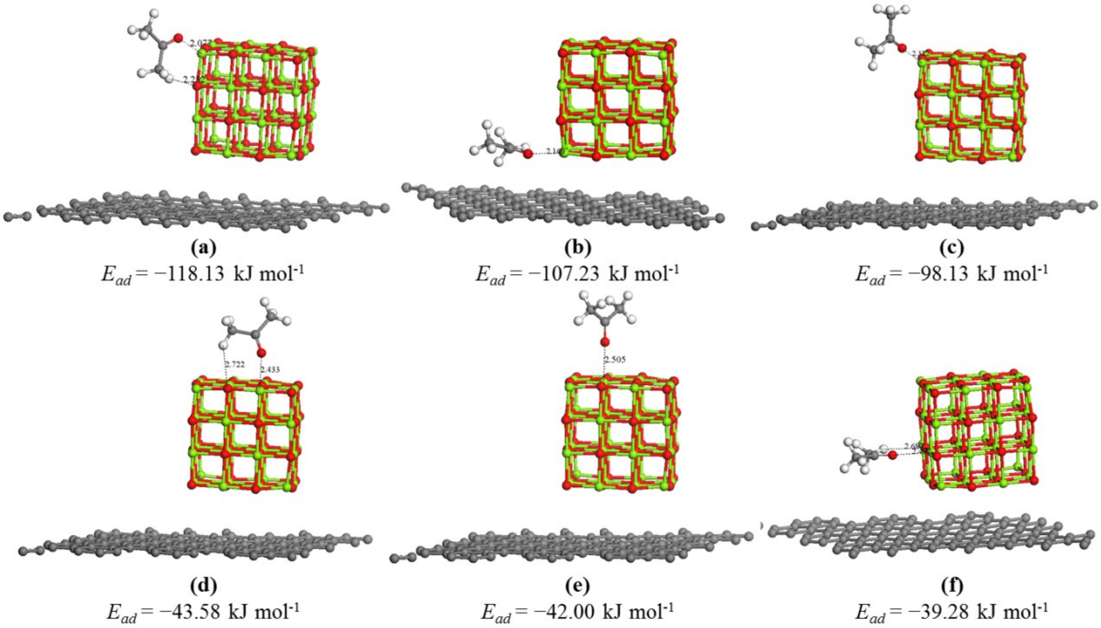

Figure S3. Molecular interaction of acetone with MgO/graphene surface.
